# Plasma copeptin is coupled to resting-state frontoinsular-subgenual alpha dynamics in major depression with comorbid post-traumatic stress disorder

**DOI:** 10.3389/fendo.2026.1895698

**Published:** 2026-09-03

**Authors:** Hiroe Hu, Benjamin H. Chandler, Jessica L. Sloane, Joseph G. Verbalis, Carlos A. Zarate, Elizabeth D. Ballard, Jessica R. Gilbert

**Affiliations:** 1 Experimental Therapeutics and Pathophysiology Branch, National Institute of Mental Health, National Institutes of Health, Bethesda, MD, United States; 2 Department of Endocrinology, Georgetown University, Washington, DC, United States

**Keywords:** anterior insula, copeptin, depression, magnetoencephalography, PTSD, sgACC, vasopressin

## Abstract

**Background:**

Hypothalamic-pituitary-adrenal axis dysregulation is implicated in depression and post-traumatic stress disorder (PTSD), but stress-related neuroendocrine mechanisms that distinguish their comorbid presentation remain poorly understood. Prior work in the broader parent cohort found lower levels of plasma copeptin, a surrogate marker of arginine vasopressin, in 0individuals experiencing a major depressive episode (MDE) who also had PTSD. The present study examined whether copeptin was associated with frequency-specific resting-state neural activity and circuit dynamics in an overlapping magnetoencephalography (MEG) subset of participants with MDE+PTSD, MDE without PTSD, and healthy volunteers (HVs).

**Methods:**

Whole blood samples were collected from 105 participants (MDE+PTSD=18, MDE=59, HV=28) and assayed for plasma copeptin concentrations. Eyes-closed resting-state MEG data were acquired and source localized using synthetic aperture magnetometry in the delta, theta, alpha, beta, gamma, and high gamma frequency bands. Linear mixed models were used to examine copeptin-by-diagnosis interactions in whole-brain source-localized power. Two regions of interest implicated in affective, interoceptive, and stress-related processing—the left anterior insula (AI) and subgenual anterior cingulate cortex (sgACC)—were further examined using virtual electrodes and dynamic causal modeling. Parametric empirical Bayesian analysis modeled group differences with mean-centered copeptin values entered as covariates.

**Results:**

In the alpha frequency band, a significant group-by-copeptin interaction (pCLC<0.05) was identified in medial and lateral prefrontal cortex, including the sgACC and AI. Compared with the HV and MDE groups, MDE+PTSD participants demonstrated a negative association between alpha power and copeptin levels. In the high gamma band, a significant main effect of diagnosis was identified in early visual cortex, encompassing the bilateral lingual gyrus, with both clinical groups showing greater power than HVs. Dynamic causal modeling of sgACC-AI circuitry showed strong evidence (Pp>0.99) for reduced intrinsic excitatory drive within the sgACC and AI in the MDE+PTSD group. Higher copeptin levels in the comorbid group were associated with local circuit changes consistent with reduced excitatory throughput and faster inhibitory timing.

**Conclusion:**

These findings raise the possibility that MDE+PTSD may represent a distinct neuroendocrine-neurophysiologic phenotype in which the relationship between peripheral vasopressinergic signaling and cortical alpha dynamics/frontoinsular-subgenual circuit function is altered.

## 1 Introduction

Major depressive episodes (MDEs) are disabling clinical states that occur across mood disorder diagnoses, including both major depressive disorder (MDD) and bipolar disorder (BD). Post-traumatic stress disorder (PTSD), another debilitating condition whose symptoms overlap significantly with those of MDEs, can precede and alter the course of MDEs (1). Because the present sample included major depressive episodes occurring in both MDD and BD, the term MDE is used throughout for the current study sample; when referring to prior literature, the diagnostic terminology used in the original reports was retained (e.g., MDD+PTSD).

MDEs with PTSD comorbidity (MDE+PTSD) often exhibit greater clinical severity than either diagnosis alone, which impacts both prognosis and public health (2). For example, in combat-related PTSD, comorbid MDD (MDD+PTSD) was linked to higher rates of suicidal ideation than PTSD alone (3). MDD+PTSD is also associated with roughly three times the rate of disability versus MDD alone, as well as with greater physical health morbidity (4). The MDE+PTSD symptom cluster is perhaps not merely additive but may reflect distinct pathophysiology. Although MDEs associated with MDD and BD share similar clinical features and patterns of brain activity across diagnoses (5), individuals with MDE and those with MDE+PTSD appear to have hyperconnectivity in the frontoparietal network (6).

Neuroendocrine endophenotypes involving the hypothalamic-pituitary-adrenal (HPA) axis and glucocorticoid receptor system have been independently studied in MDD, BD, and PTSD (7–11), given that MDEs associated with MDD, BD, and PTSD all involve a stress-related etiology to varying degrees (12). Though stress-related mechanisms that distinguish PTSD comorbidity during MDEs remain relatively under-characterized, preliminary evidence suggests that the comorbid condition represents a distinct neuroendocrine biotype compared to mono-diagnostic conditions. One study found that individuals with MDD+PTSD compared to PTSD alone had an attenuated HPA axis response (13). In addition, a previous study from our laboratory found that MDE+PTSD participants had attenuated arginine vasopressin (AVP) secretion compared to those with MDD or BD who had mono-diagnostic MDEs (14). AVP is a key neurohormone regulator of the HPA axis stress response (15, 16) and has been implicated in the pathophysiology of mood disorders (17–19), PTSD (20), as well as key markers of clinical severity like suicide risk (21, 22) and treatment resistance (23).

Compared to cortisol-focused models of mood disorders and PTSD, AVP has been historically understudied in large part due to measurement barriers (17). The methodological sophistication of neuropeptide measurement, however, has grown in the last decade, owing to the use of copeptin as a surrogate marker for AVP. Copeptin is the c-terminal portion of the precursor pre-prohormone of AVP; compared to AVP, it has a longer half-life, greater stability, and larger peptide size for ease of measurement. Copeptin is secreted with AVP in equimolar ratio, ensuring the close approximation of cumulative AVP levels (24). Our prior results of blunted AVP secretion in MDE+PTSD participants compared to those experiencing an MDE without comorbid PTSD and healthy volunteers (HVs) were also characterized successfully using copeptin as a surrogate peripheral marker for AVP. A major limitation in solely using peripheral neuroendocrine biomarkers, however, is the absence of well-defined neural correlates indicating that the peripheral levels have central engagement. Neuroimaging studies of exogenously (intranasally) administered AVP are therefore critical for establishing that neuropeptide systems can alter human brain function while also highlighting ongoing uncertainty about central nervous system penetrance through intranasal administration and the highly context-dependent nature of AVP’s action on the brain (25).

In this context, a critical need exists to link peripheral endogenous AVP levels (using copeptin as a surrogate marker) with neural biomarkers, a strategy that could help define clinically meaningful subtypes involving mood disorders and PTSD. Magnetoencephalography (MEG) is well suited for linking peripheral neuroendocrine markers with cortical dynamics because it measures magnetic fields generated by the electrical activity of synchronized neuronal populations and supports frequency-specific analysis of source-localized cortical power—features that are difficult to infer from hemodynamic signals alone (26). MEG is also a compelling method for interrogating frequency-specific neural signatures in MDE+PTSD, compared to MDE or in HVs, that correspond with peripheral copeptin levels. MEG is also uniquely suited to uncovering mechanistic principles because it can help bridge animal and computational models with human data (26).

The frequency-specificity of MEG is of particular interest considering that patients with PTSD and depression often show opposing alpha-band patterns—a candidate frequency for this investigation. Alpha oscillations, which are the dominant rhythm at rest, are associated with default mode network (DMN) activity (27) and, inversely, with cortical excitability (28). In PTSD, convergent electrophysiological evidence suggests reduced alpha power and alpha dysrhythmia, including in DMN hubs, with symptom associations that implicate hypervigilance and sensory disinhibition (29, 30). In contrast, MDD is often characterized by increased global alpha power and altered laterality/connectivity patterns (31, 32). Electrophysiologic evidence in the comorbid subgroup is far more limited; however, one resting-state electroencephalography (EEG) study in individuals with comorbid PTSD and MDD suggested that alpha-band coherence may carry clinical relevance in this population (33).

Despite extensive work on alpha oscillations in PTSD and emerging evidence for vasopressinergic dysregulation in mood and stress-related disorders, no studies to date have examined the relationship between peripheral AVP activity and frequency-specific source-localized power in individuals with comorbid depression and PTSD. Interestingly, several lines of evidence motivate a focus on medial prefrontal and frontoinsular circuitry—systems repeatedly implicated in both depressive and trauma-related phenotypes, with particular attention to two regions of interest (ROIs): the subgenual anterior cingulate cortex (sgACC) and the anterior insula (AI). The sgACC is one of the central nodes in mood disorders pathophysiology. Preclinical and translational models indicate that the sgACC participates in an extended visceromotor network that modulates autonomic and neuroendocrine responses during reward, fear, and stress processing (34)—precisely the functional domains implicated at the intersection of depression and PTSD. The AI, a salience network hub, supports interoception and salience detection and has been linked to clinically meaningful electrophysiologic signatures in MDEs (35–37) as well as in PTSD (38–40). Postmortem anatomical work identified AVP-immunoreactive fibers in human cortical regions including the anterior cingulate cortex (ACC) and insula (41), suggesting a plausible substrate for vasopressinergic modulation of cortical function in regions central to affect and interoceptive regulation. Complementing this, intranasal AVP has been shown to modulate sgACC activation and medial prefrontal cortex-amygdala circuitry during emotion processing (42).

This study sought to investigate resting-state neural correlates of vasopressinergic mechanisms indexed by peripheral copeptin, specifically whether copeptin levels are associated with whole-brain, frequency-specific resting-state power and whether these relationships differ across MDE+PTSD, MDE, and HV groups. Given prior evidence of blunted vasopressinergic signaling in MDE+PTSD, opposing alpha-band abnormalities reported in PTSD and depression, and the central role of sgACC-AI circuitry in stress-related psychopathology, it was hypothesized that copeptin would show diagnosis-dependent associations with alpha power, particularly in medial prefrontal and frontoinsular regions relevant to salience and interoceptive processing. Individuals with MDE+PTSD were hypothesized to exhibit an altered copeptin-alpha relationship relative to HVs and to those experiencing an MDE without comorbid PTSD, further raising the possibility of a distinct neuroendocrine-neurophysiologic subtype. To test whether these effects extended beyond regional power to circuit-level dynamics, dynamic causal modeling (DCM) was used to model effective connectivity between the sgACC and AI.

## 2 Materials and methods

### 2.1 Study participants and procedures

Biological samples and neuroimaging data were collected from 151 males and females (N=151; aged 18–70 years) enrolled in the inpatient Neurobiology of Suicide protocol (NCT02543983) at the Mood Disorders Research Unit of the National Institute of Mental Health (NIMH), National Institutes of Health (NIH), Bethesda, Maryland, between 2015 and 2023. All participants completed a comprehensive screening protocol (NCT00024635) prior to enrollment. The primary goal of the parent study was to characterize peripheral and neuroimaging biomarkers of suicide risk; detailed information regarding recruitment procedures, suicide risk stratification, and inclusion and exclusion criteria is provided in the Supplement. All participants provided written informed consent prior to study entry, and all procedures were approved by the NIH Combined Neuroscience Institutional Review Board.

The present study leveraged the protocol’s broad inclusion criteria, which sought to recruit individuals experiencing a current MDE across varying levels of suicidality and heterogeneous psychiatric comorbidities, including PTSD. Psychiatric diagnoses were established using the Structured Clinical Interview for DSM Disorders (43) (SCID) administered during the screening protocol. Participants with current psychotic features, cognitive impairment, substance dependence, or unstable medical conditions were excluded from both the parent protocol and the present analyses. Clinical assessments administered as part of the inpatient protocol were used in the present study. These included the Montgomery-Åsberg Depression Rating Scale (MADRS) (44) and the Impact of Event Scale–Revised (45) (IES-R), which assesses current trauma-related distress.

Whole blood samples were collected from participants in the morning using BD Vacutainer heparinized tubes and centrifuged at 3,000 rpm for 10 minutes at 4°C. Plasma was separated, aliquoted, and stored at −80°C until assay. Plasma copeptin concentrations were quantified at Mayo Clinic Laboratories (Rochester, MN) using the automated B·R·A·H·M·S Copeptin proAVP KRYPTOR immunofluorescent assay on the KRYPTOR Compact PLUS platform. The lower limit of detection was 2.9 pmol/L, and reference intervals are <13.1 pmol/L for non-water deprived healthy adults and <15.2 for adults who are water-deprived for at least 8 hours (which was not the case for our participants). Laboratory-specific intra-assay and inter-assay coefficients of variation were not provided in the assay report and were therefore unavailable for the present study.

### 2.2 MEG acquisition and preprocessing

A subset of participants (N=105) also completed resting-state MEG scans during their inpatient admission. MEG data were acquired during a session temporally proximal to biological sample collection and clinical assessments. Only participants with available MEG data and corresponding copeptin measurements were included in the present analysis. For participants included in MEG analyses, the absolute interval between blood collection and MEG acquisition ranged from 0 to 7 days, with a mean of 0.60 days (SD=1.25) and a median of 0 days (IQR=0–1). Sixty-seven participants (63.8%) completed both on the same day, and 98 participants (93.3%) completed them within one day; the full distribution is provided in **Supplementary Table 1**.

Neuromagnetic data were recorded at 1200Hz using a bandwidth of 0.61–300 Hz. Data were collected in a magnetically shielded room using a SQUID-based CTF 275 system. One or two 480-second resting-state MEG recordings were obtained during the scan session, and participants were instructed to remain as still as possible with their eyes closed. For those participants with multiple rest scans, the first scan was acquired at the beginning of the scan session, and the second scan was acquired following completion of a series of tasks. Synthetic third-order balancing was used for active noise cancellation. In addition to MEG recordings, a T1-weighted MRI scan was collected using a 3 Tesla GE scanner and used for data coregistration using fiducial landmarks placed at the nasion and left and right preauricular locations digitized during MEG recordings.

This work used the computational resources of the NIH HPC Biowulf cluster (https://hpc.nih.gov). Data were initially bandpass filtered from 1–58 Hz for cleaning, and the fast ICA algorithm was used to identify potential cardiac, ocular, and movement artifacts using MNE-python templates (46). Following visual inspection, identified artifacts were removed from subsequent analysis. In addition, sensors that included significant artifacts or excessive noise were removed from the analysis.

### 2.3 Source analysis

Cleaned data were localized to source space using a 5-mm grid and a semi-realistic head model (47) using synthetic aperture magnetometry (SAM) (48). Beamformer weights were calculated using a bandpass frequency of 2 to 100Hz, and power was normalized by the projected noise floor of the virtual sensor. Power was projected in the following frequency bands: delta (2–4 Hz), theta (4–8 Hz), alpha (8–14 Hz), beta (14–30 Hz) gamma (30–58 Hz), and high gamma (62–100 Hz). Resulting power images were warped to Talairach space and masked to remove non-brain matter and the cerebellum using Analysis of Functional NeuroImages (AFNI) (49). Final root-mean-square power images were calculated by normalizing masked images by the square root of the sum of the squared images for the six canonical bands between 2–100 Hz.

Linear mixed models were constructed using the 3dLMEr routine in AFNI (50). Factors included in the model were copeptin level, diagnosis, scan order, and sex, with all two-, three-, and four-way interactions considered. Age was included additively in the model formula to adjust for its effects. The specific focus was on main effects of diagnosis and copeptin levels, as well as diagnosis-by-copeptin interactions. Initial statistical maps were first thresholded at a voxel-wise level of p<0.01 (uncorrected), and AFNI’s 3dClustSim program was used to evaluate clusters that achieved cluster-level significance of p<0.05 FWE-correction. Separate models were run for each of the six frequency bands of interest.

Exploratory sensitivity analyses evaluated whether the primary diagnosis-by-copeptin interaction was materially altered by depressive symptom severity (MADRS), trauma-related symptom severity (IES-R), lithium and diuretic use, the interval between blood collection and MEG acquisition, and available physiological variables relevant to copeptin regulation, such as plasma osmolality and renal function. Given the modest subgroup sizes and collinearity among some covariates, these factors were examined in separate models rather than simultaneously.

Two ROIs—the sgACC and AI—were probed to examine connectivity differences between groups, based on their established roles in depression and stress-related pathophysiology (34, 51). These literature-motivated ROIs also overlapped with the diagnosis-by-copeptin interaction observed in the alpha frequency in the whole-brain source analysis, providing additional support for examining sgACC–AI effective connectivity in secondary DCM analyses. Source activity was extracted from these regions by constructing virtual sensors with target locations in left AI (Talairach coordinates: -30, 18, 0) and sgACC (Talairach coordinates: -3, 31, -1). These timeseries estimates were imported into SPM12 (http://www.fil.ion.ucl.ac.uk/spm/) and epoched into two-second trial lengths for subsequent connectivity modeling.

### 2.4 Dynamic causal modeling

DCM uses biophysical models of brain dynamics and neural mass models to predict electrophysiological data features. The present study used the “CMM_NMDA” model to examine differences in sgACC-AI connectivity between groups. The model includes parameters for both fast (α-amino-3-hydroxy-5-methylisoxazole-4-propionic acid (AMPA)-mediated) and slow (N-methyl-D-aspartate (NMDA)-mediated) glutamatergic signaling. Within the model, pyramidal cells in superficial layers of the cortex encode and carry feedforward signaling to stellate cells, while deep pyramidal cells carry feedback signaling to pyramidal neurons and interneurons.

**Figure 1:**
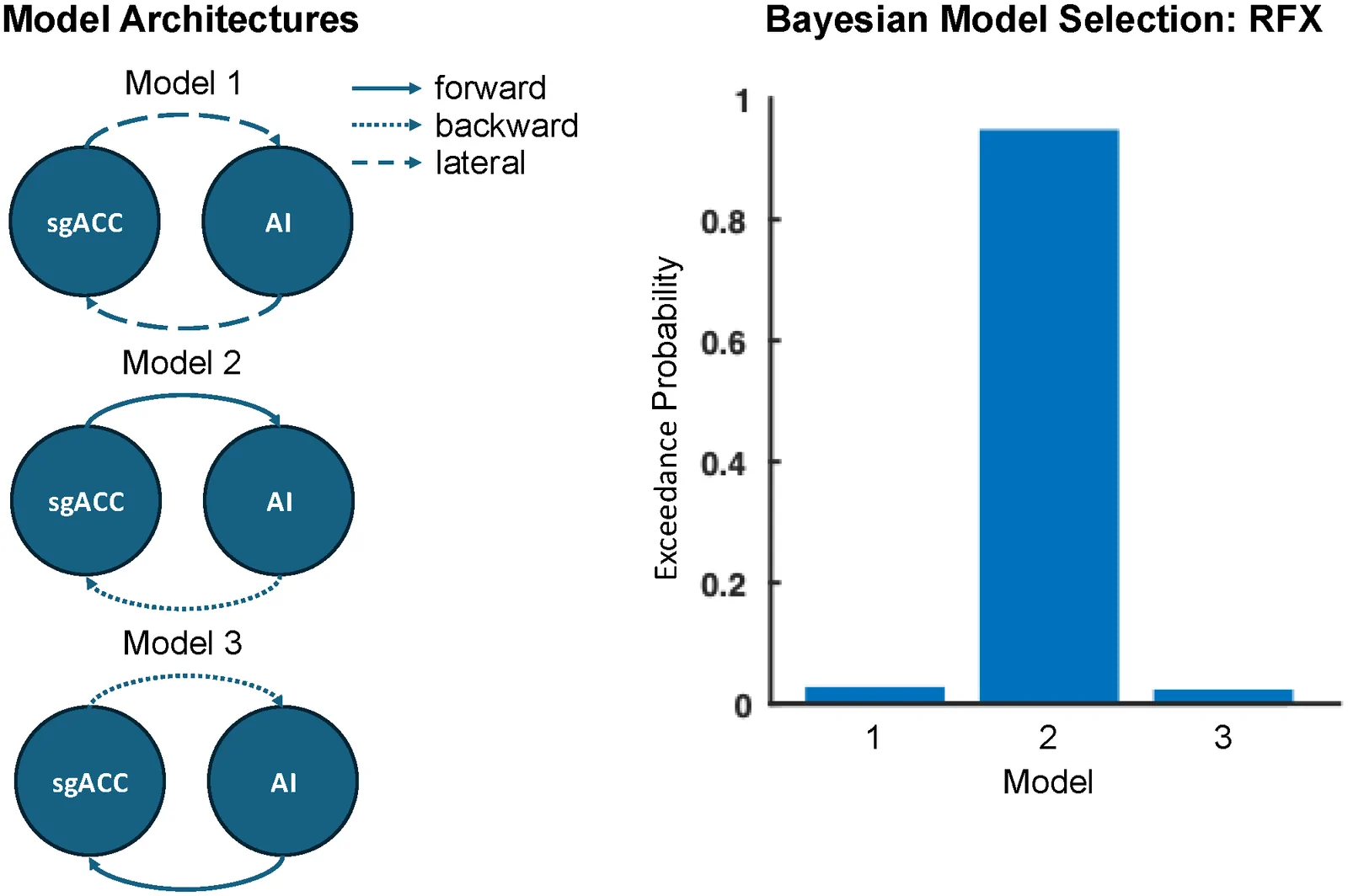
Model architectures and Bayesian model selection. Three models of message-passing between the subgenual anterior cingulate cortex (sgACC) and anterior insula (AI) were compared to test effective connectivity. Random-effects Bayesian model selection favored Model 2, supporting an asymmetric architecture with feedforward signaling from sgACC to AI and reciprocal feedback signaling from AI to sgACC.

For the DCM analyses, extracted MEG time series were modeled across all two-second resting-state epochs from 1–30 Hz using a local field potential model to estimate cross-spectral density estimates from the AI and sgACC. Using a standard variational Bayesian inversion approach, model parameters were inferred by optimizing the posterior distribution over free parameters, which were defined by their expected mean and covariance. Three models were constructed to examine message-passing between the ROIs. Model 1 included bidirectional feedforward and feedback signaling between the sgACC and the AI. Model 2 included feedforward connections from the sgACC to the AI, with reciprocal feedback connections from the AI to the sgACC. Model 3 included feedforward connections from the AI to the sgACC, with reciprocal feedback connections from the sgACC to the AI (**Figure 1**). The negative free energy bound on the log-model evidence was used to adjudicate between models. Parameter estimates were then extracted from the optimized DCMs for the winning model separately for each subject and session to evaluate group differences.

Parametric empirical Bayesian (PEB) analysis (52), a second-level modeling extension of DCM, was used to model differences between groups. The first column of the analysis tested for the average effect across all participants, while the second column tested for the difference between the MDE+PTSD group and the average effect. The third column included mean-centered copeptin values, while the fourth column tested the MDE+PTSD-by-copeptin interaction. This analysis refits a full model, allowing all parameters to covary according to grouping factors and provides reduced models in which smaller combinations of parameters are considered and informed by differences between groups and covariates of interest. This analysis considered the following factors: parameters governing time constants of AMPA, NMDA, and gamma aminobutyric acid (GABA); the membrane capacitance of superficial and deep pyramidal cells, spiny stellate cells, and inhibitory interneurons; and AMPA- and NMDA-mediated intrinsic and extrinsic connectivity.

## 3 Results

### 3.1 Participant characteristics

The final MEG sample included 105 participants: 28 HVs, 59 participants experiencing an MDE without PTSD (the MDE group), and 18 participants with comorbid PTSD and MDE (MDE+PTSD). Demographic characteristics were generally comparable across groups, and no significant group differences were observed with regard to sex distribution, race, or ethnicity; age showed only a trend-level difference across groups (p=0.065). Clinical characteristics differed in expected ways. Total medication trials differed significantly across groups (p<0.001), with HVs having no prior medication trials and both clinical groups showing greater medication exposure. Severity of depressive symptoms also differed significantly across groups (MADRS: p < 2 × 10^-16), driven primarily by markedly lower scores in HVs relative to both patient groups. Trauma-related symptom severity also differed significantly across groups (IES-R: p=1.61 × 10^-9), with the highest scores observed in the MDE+PTSD group.

Plasma copeptin levels did not differ significantly across groups in this MEG subset (p=0.444), although mean copeptin levels were numerically lower in the MDE+PTSD group than in the MDE and HV groups. The absolute interval between blood collection and MEG acquisition did not differ significantly across groups, nor did plasma sodium, osmolality, or creatinine levels (all *p*>.10; **Table 1**).

**Table 1:** Demographics and clinical data.

Participant Characteristics	Total (N=105)	HV (N=28)	MDE (N=59)	MDE+PTSD (N=18)
Participants				
Female (%)	59 (56.19)	14 (50.00)	33 (55.93)	12 (66.67)
Mean Age (SD)	42.64 (14.74)	38.53 (13.90)	42.31 (14.41)	45.26 (15.78)
Race				
American Indian/Native (%)	2 (1.9)	0 (0.00)	2 (3.39)	0 (0.00)
Asian (%)	10 (9.52)	4 (14.29)	6 (7.23)	2 (5.88)
Black/African American (%)	17 (12.87)	7 (25.00)	9 (15.25)	2 (11.11)
Multiple Races (%)	4 (3.81)	1 (3.57)	2 (3.39)	1 (5.56)
Other (%)	2 (1.90)	0 (0.00)	1 (1.20)	2 (11.11)
Unknown (%)	2 (1.90)	0 (0.00)	1 (1.69)	1 (5.56)
White (%)	67 (63.81)	16 (57.14)	40 (67.80)	11 (61.11)
Ethnicities				
Latino or Hispanic (%)	8 (7.62)	3 (10.71)	2 (3.39)	3 (16.67)
Not Latino or Hispanic (%)	92 (87.62)	25 (89.29)	54 (91.53)	13 (72.22)
Unknown (%)	5 (4.76)	0 (0.00)	3 (5.08)	2 (11.11)
Clinical characteristics				
Bipolar Disorder (%)	17 (16.19)	0 (0.00)	12 (20.34)	5 (27.78)
Antidepressant Use (%)	44 (41.90)	0 (0.00)	36 (61.02)	8 (44.44)
Lithium Use (%)	4 (3.81)	0 (0.00)	2 (3.39)	2 (11.11)
Diuretic Use (%)	5 (4.76)	0 (0.00)	4 (6.78)	1 (5.56)
Past Antidepressant Trials Mean (SD)	1.5(2.23)	0 (0.00)	1.9 (2.17)	2.5 (3)
MADRS [0 – 60], Mean (SD)	15.9 (13.04)	0.57 (0.96)	21.71 (10.67)	20.72 (11.01)
IES-R [0 – 88], Mean (SD)	11.22 (11.82)	1.11 (3.18)	13.05 (11.15)	20.94 (11.59)
Interval between MEG and Blood Collection, Mean (SD)	0.60 (1.25)	0.32 (0.48)	0.76 (1.43)	0.50 (1.42)
Biological variables				
Plasma Copeptin, Mean (SD)	5.23 (4.85)	5.62 (4.81)	5.45 (4.7)	3.91 (5.44)
Plasma Sodium, Mean (SD)	140.21 (2.05)	139.93 (2.14)	140.29 (2.02)	140.39 (2.09)
Plasma Osmolality, Mean (SD)	290.40 (4.58)	289.96 (5.23)	290.62 (4.24)	290.40 (4.83)
Plasma Creatine, Mean (SD)	0.83 (0.16)	0.79 (0.16)	0.85 (0.17)	0.83 (0.15)

HV, healthy volunteer; IES-R, Impact of Event Scale-Revised; MADRS, Montgomery-Asberg Depression Rating Scale; MDE, major depressive episode; PTSD, post-traumatic stress disorder. MADRS is measured on a scale of 0-60, and IES-R is measured on a scale of 0-88. Intervals between MEG and blood collection are reported in absolute numbers of days. Units of biological variables: plasma Copeptin in pmol/L, plasma sodium in mmol/L, plasma osmolality in mOsm/kg, plasma creatinine in mg/dL.

When restricting comparisons to the two clinical groups, the MDE and MDE+PTSD groups did not differ significantly in terms of total medication trials, MADRS scores, copeptin levels, current antidepressant use, rate of BD, lithium use, or diuretic use. As expected, IES-R scores remained significantly higher in the MDE+PTSD group (p=0.011). Additional demographic and clinical characteristics are summarized in **Table 1**.

### 3.2 Source-level

**Figure 2:**
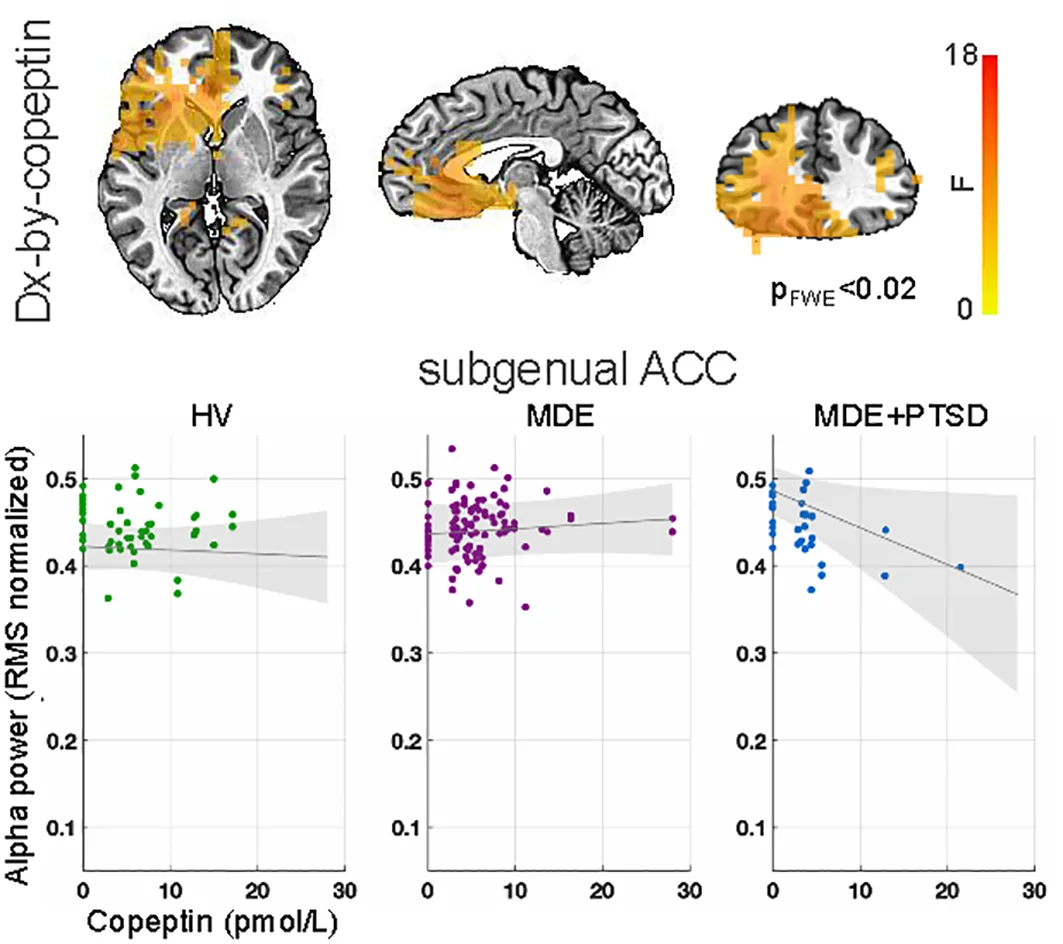
Alpha diagnosis-by-copeptin sinteraction. Brain regions demonstrating a group-by-copeptin interaction in the alpha (8–14 Hz) frequency are superimposed on a template brain (top). The significant cluster encompassed broader frontoinsular and medial prefrontal regions, including the anterior insula (AI) and subgenual anterior cingulate cortex (sgACC). The color bar represents the magnitude of the F statistic. To illustrate the direction of the interaction at a clinically relevant location within this cluster, mean alpha power and copeptin levels are plotted for the sgACC (bottom). Compared with healthy volunteers (HVs) and participants experiencing a major depressive episode (MDE) without post-traumatic stress disorder (PTSD), participants with MDE+PTSD showed a strong negative association between alpha power and copeptin at the sgACC location.

Linear mixed effects models were used to examine the relationship between copeptin levels and resting-state source-localized power in the delta, theta, alpha, beta, gamma, and high gamma frequencies in the three groups. Two principal findings emerged that survived cluster-level correction. First, in the alpha frequency, there was a significant diagnosis-by-copeptin interaction that encompassed ventromedial and ventrolateral prefrontal cortical regions as well as the AI and sgACC (cluster-extent threshold of k>104.8 voxels, cluster-level significance of p<0.05 FWE-corrected, Talairach peak=-22, 23, -8). In examining the interaction, the MDE+PTSD group demonstrated a strong negative association between alpha power and copeptin levels. In contrast, the MDE group demonstrated a slightly positive association between alpha power and copeptin levels, and the HV group demonstrated no association between alpha power and copeptin levels (**Figure 2**). There was no significant main effect of sex or sex-related interactions.

Based on the small sample size of MDE+PTSD participants, leave-one-out (LOO) cross-validation was performed within the MDE+PTSD group by rerunning the model 18 times, each time excluding one participant. For each fold, the group-level alpha power estimate was extracted within the ROI plotted in **Figure 2**. The LOO estimates were highly stable (mean alpha power=9.79, SD=1.03), with all folds preserving the sign of the effect and showing modest deviations from the full-sample estimate. The 95% confidence interval across LOO folds (7.77–11.81) excluded zero, indicating that the observed effect was not driven by any single participant. These results demonstrate strong robustness of the group-level effect under subject-wise removal.

Exploratory models including MADRS and IES-R entered additively as covariates did not materially alter the copeptin-by-diagnosis findings, suggesting that the observed neural signatures were not explained solely by depressive or trauma-related symptom severity in this sample. In medication-related sensitivity analyses, the diagnosis-by-copeptin interaction remained significant after excluding the four participants currently taking lithium. After excluding the five participants currently taking diuretics, a spatially overlapping interaction remained evident but no longer survived cluster-level correction (**Supplementary Figure 1**).

**Figure 3:**
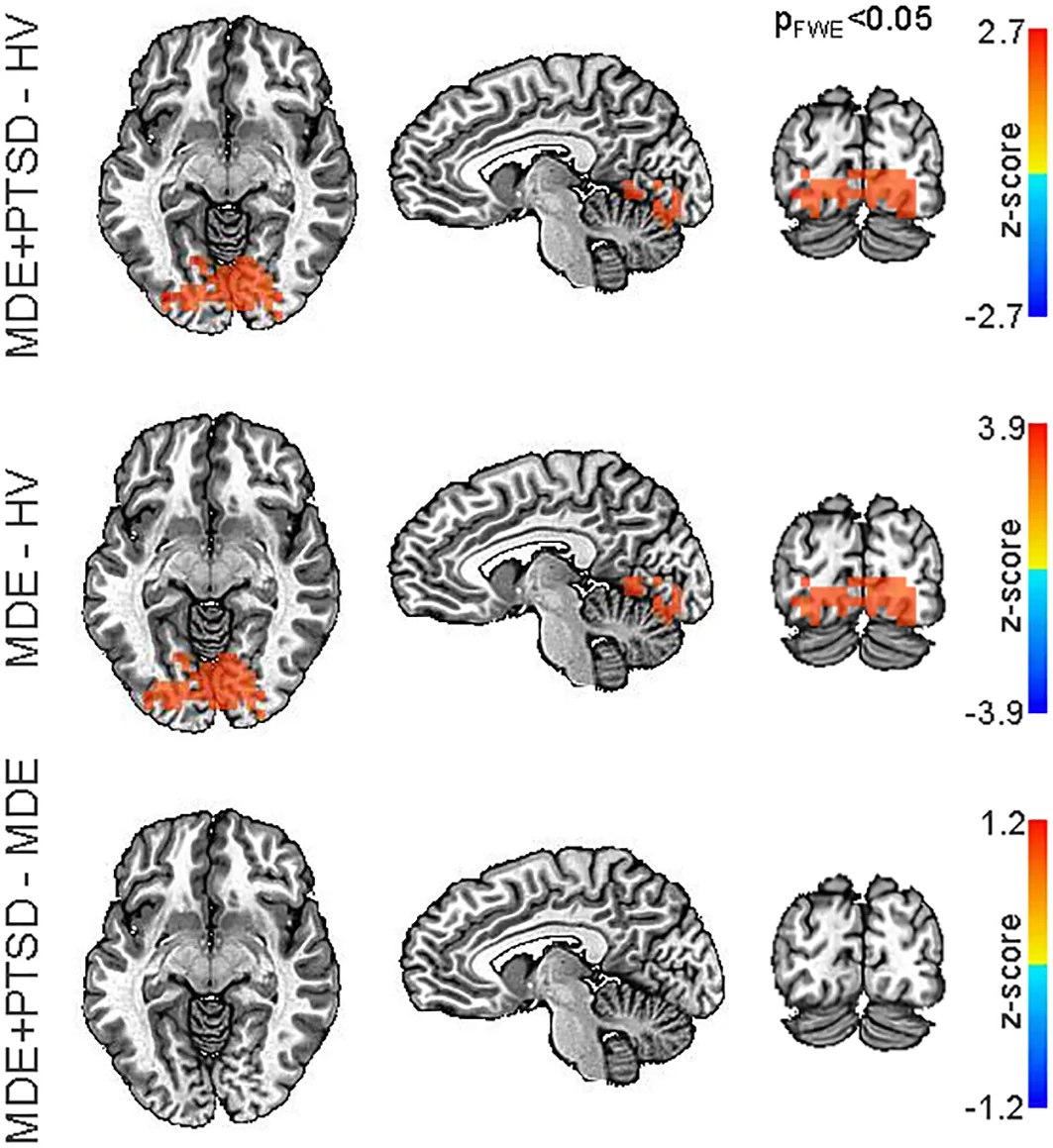
High gamma diagnosis main effect. Brain regions demonstrating a main effect of diagnosis in the high gamma frequency (62–100 Hz) are superimposed on a template brain. The significant cluster was localized to early visual cortex and encompassed the bilateral lingual gyrus. *Post-hoc* contrasts indicated that both the major depressive episode+post-traumatic stress disorder (MDE+PTSD) and MDE groups exhibited stronger high gamma power than healthy volunteers (HVs) in this region, whereas no significant difference was observed between the MDE+PTSD and MDE groups. The color bars represent z-scores for each pairwise contrast. PTSD, post-traumatic stress disorder; MDE, major depressive episode.

Second, in the high gamma frequency, a significant main effect was noted for diagnosis in the early visual cortex that encompassed the bilateral lingual gyrus (cluster-extent threshold of k>99.5 voxels, cluster-level significance of p<0.05 FWE-corrected). Both the MDE and MDE+PTSD groups exhibited stronger high gamma power compared to the HV group in this region (**Figure 3**).

### 3.3 Dynamic causal modeling

As a secondary, model-based follow-up to the source-level alpha findings, three candidate models of message-passing between the AI and sgACC were constructed. Bayesian model selection was used to compare the models using the negative free energy bound on the log-evidence. Model 2, which included feedforward connections from the sgACC to the AI with reciprocal feedback connections from the AI to the sgACC, was identified as having the highest posterior probability (Model 1 exceedance probability=0.0278, Model 2 exceedance probability=0.9487, Model 3 exceedance probability=0.0235) (**Figure 1**). The average variance explained by Model 2 was 90.2% across participants.

All fitted parameters from Model 2 were extracted and a parametric empirical Bayesian approach was used to evaluate group-level effects on the estimated model parameters. This approach was used to test for the average effects across all participants, the effects of MDE+PTSD compared to the group mean, the effects of copeptin, and copeptin-by-MDE+PTSD interactions. Parameters having a posterior probability ≥ 0.99 were defined as exhibiting very strong evidence for an effect. Complete parameter results are provided in **Tables 2**, **3**, and the group effects exhibiting very strong evidence are summarized below. Our second-level analysis of group effects identified nine meaningful parameters that differentiated the MDE+PTSD group from the group mean. These included two parameters governing time constants for GABA in both the sgACC (Ep=-0.135, Pp=1) and the AI (Ep=-0.2984, Pp=1). In addition, one parameter governing the membrane capacitance of inhibitory interneurons (Ep=0.3646, Pp=1) across the model was identified. Intrinsically, parameters governing connectivity between spiny stellate cells and superficial pyramidal cells in both the sgACC (Ep=-0.3455, Pp=1) and the AI (Ep=-0.702, Pp=1), as well as self-connections on both superficial pyramidal cells and inhibitory interneurons in both the sgACC (sp-self Ep=0.2357, Pp=1; ii-self Ep=-0.3775, Pp=1) and the AI (sp-self Ep=0.4362, Pp=1, ii-self Ep=-0.254, Ep=1) were altered in the MDE+PTSD group compared to the MDE and HV groups.

**Table 2:** Group effects over parameters.

#	Parameter	Parameter estimate (Ep)	Posterior probability (Pp)
Time constants			
1	GABA – sgACC	-0.135	1*
2	GABA – AI	-0.2984	1*
3	NMDA – sgACC	0.1133	0.6003
Membrane capacitance			
4	ii*	0.3646	1*
Extrinsic connectivity			
5	AMPA – sgACC to AI	0.2244	0.5746
6	Intrinsic Connectivity	-0.3455	1*
7	sgACC – ss to sp	-0.702	1*
8	AI – ss to sp	0.2357	1*
9	sgACC – sp self-connection	0.4362	1*
10	AI – sp-self	-0.3775	1*
11	sgACC – ii self-connection AI – ii self-connection	-0.254	1*

Parametric empirical Bayes was used to identify the mixing of parameters contributing to MDE+PTSD group effects. Meaningful parameters were defined as those with posterior probability (Pp) > 0.99*. MDE, major depressive episode; PTSD, post-traumatic stress disorder; sgACC, subgenual anterior cingulate cortex; AI, anterior insula; GABA, gamma-aminobutyric acid; NMDA, N-methyl-D-aspartate; AMPA, α-amino-3-hydroxy-5-methyl-4-isoxazolepropionic acid; ii, inhibitory interneurons; ss, spiny stellate cells; sp, superficial pyramidal cells.

**Table 3:** Group by copeptin effects over parameters.

#	Parameter	Parameter estimate (Ep)	Posterior probability (Pp)
Membrane capacitance			
1	sp	0.0631	1*
2	ii	-0.0819	1*
Intrinsic connectivity			
3	sgACC – ss to sp	-0.1132	1*
4	AI – ss to sp	-0.0726	1*
5	sgACC – ii self-connection	-0.0723	1*
6	sgACC – sp self-connection	0.0289	0.5936

Parametric empirical Bayes was used to identify the mixing of parameters contributing to MDE+PTSD-by-copeptin interaction effects. Meaningful parameters were defined as those with posterior probability (Pp) > 0.99*. MDE, major depressive episode; PTSD, post-traumatic stress disorder; sgACC, subgenual anterior cingulate cortex; AI, anterior insula; ii, inhibitory interneurons; ss, spiny stellate cells; sp, superficial pyramidal cells.

Our second-level analysis of parameters demonstrating MDE+PTSD-by-copeptin interaction effects identified five meaningful parameters using a posterior probability ≥ 0.99. Two parameters governing the membrane capacitance of both superficial pyramidal cells (Ep=0.0631, Pp=1) and inhibitory interneurons (Ep=-0.0819, Pp=1) across regions demonstrated interaction effects. In addition, intrinsically, parameters governing connectivity between spiny stellate cells and superficial pyramidal cells in both the sgACC (Ep=-0.1132, Pp=1) and the AI (Ep=-0.0726, Pp=1), as well as the self-connections on inhibitory interneurons (Ep=-0.0723, Pp=1) in the sgACC demonstrated interaction effects.

## 4 Discussion

This study addresses two important gaps in the literature by linking plasma copeptin, a marker of neurohypophyseal AVP secretion, to resting-state MEG and effective connectivity measures and by directly examining the comorbid MDE+PTSD subgroup relative to MDE and HVs. The most pronounced MDE+PTSD effects emerged in the alpha frequency band, where the comorbid group showed a strong inverse association between copeptin levels and source-localized alpha power in frontoinsular and medial prefrontal regions, particularly the sgACC and AI. These findings raise the possibility that comorbid MDE+PTSD may involve a distinct pattern of copeptin–neural coupling in which the relationship between peripheral vasopressinergic signaling and cortical network dynamics is altered. Given the modest size of the MDE+PTSD subgroup, however, the present findings are best viewed as exploratory and hypothesis-generating, warranting replication in larger and more balanced samples.

Importantly, although our prior study in the broader parent cohort found lower basal copeptin in MDE+PTSD (14), the present MEG subset (a smaller sample) found no significant between-group difference in copeptin. Accordingly, the present findings are best interpreted as evidence of an altered copeptin–brain relationship in the comorbid subgroup rather than direct evidence that lower copeptin levels explain altered brain function. Nonetheless, in examining the relationships between source-localized power and copeptin levels across the MDE+PTSD, MDE, and HV groups, two frequency-specific findings emerged.

First, relative to HVs, individuals experiencing an MDE showed greater high gamma power in early visual cortex regardless of PTSD comorbidity. Given that this effect was not related to copeptin, it may represent a shared neural feature of depressive episodes more broadly. Prior electrophysiologic work has similarly suggested that mood disorders involve abnormalities across higher frequency activity and large-scale cortical organization, including altered gamma-related and cross-frequency patterns in depression (35, 53). In this context, the high gamma result may index a diagnosis-related shift in cortical excitation-inhibition balance during MDEs that is distinct from the copeptin-linked effects observed in the alpha band.

Second, the only frequency in which the relationship between neural power and copeptin varied as a function of diagnostic group was the alpha band. Rather than reflecting a uniform association between vasopressinergic signaling and cortical activity, this pattern was specific to the comorbid subgroup, who exhibited a negative association between copeptin levels and alpha power in fronto-insular and medial prefrontal regions, including the sgACC and AI. This finding is notable given that prior work has shown that PTSD and depression often exhibit opposing alpha-band profiles, with PTSD more commonly associated with reduced alpha power and dysrhythmia (29, 30, 54), and MDD more often associated with increased global alpha power and altered laterality and connectivity patterns (31, 32). Alpha oscillations are also closely tied to inhibitory control and cortical excitability while contributing to large-scale resting-state network organization, including in the DMN (27, 28). The specificity of the copeptin-alpha association to the MDE+PTSD group but not to the MDE or HV groups suggests that this relationship is not a general feature of depressive episodes alone, though whether it extends to PTSD without comorbid depression remains an open question. The clinical relevance of alpha dynamics in related large-scale networks is further underscored by a series of randomized controlled trials of alpha-targeted EEG neurofeedback in PTSD, in which experimentally increasing alpha synchrony was associated with reduced PTSD symptoms and normalization of both DMN and salience network connectivity (55–57).

The localization of the copeptin-related alpha effect to the sgACC and AI is biologically and clinically meaningful and converges with independent lines of evidence implicating these regions at the intersection of depressive and trauma-related pathophysiology. The sgACC is a central node in mood disorder pathophysiology, showing structural and metabolic abnormalities that normalize upon clinical remission, and has been repeatedly implicated in mood regulation as well as in autonomic and neuroendocrine integration through its role in an extended visceromotor network that modulates responses during reward, fear, and stress processing (34). Complementing our comparison of MDE+PTSD versus MDE and HV participants, a resting-state fMRI study of PTSD patients with and without comorbid MDD found that sgACC-seeded functional connectivity distinguished the MDD+PTSD subgroup (58), further implicating sgACC-centered circuitry in depressive-traumatic comorbidity distinguishable from a mono-diagnostic subgroup. The AI, as a core salience network hub, supports interoception, bodily threat signaling, and dynamic attentional switching between internally and externally oriented brain states (51). These functions have been found to be disrupted in both depression (36, 37) and PTSD (39).

The extension of the cluster into adjacent ventromedial and ventrolateral prefrontal regions further situates the sgACC and AI findings within a broader triple-network framework, implicating interactions among salience, default-mode, and executive-control systems (59). Prior resting-state studies of comorbid PTSD and unipolar depression (MDD+PTSD) provide complementary evidence for altered interactions across these networks. In a comparison of individuals with PTSD alone and those with MDD+PTSD, greater salience–default-mode network connectivity helped distinguish the PTSD-alone group from the comorbid group (60). In a separate four-group study including participants with PTSD alone, MDD alone, comorbid MDD+PTSD, and HVs, the comorbid group showed increased intrinsic connectivity within the right frontoparietal network (6). Together, these findings suggest that depressive–traumatic comorbidity may involve altered coordination among salience, default-mode, and executive-control systems rather than dysfunction confined to individual regions.

Given the proposed role for the AI in salience-mediated network switching (51) and the contribution of alpha oscillations to large-scale network organization (27), the distributed copeptin–alpha relationship observed here may reflect altered coordination between internally oriented affective and visceromotor processing and externally oriented cognitive control in comorbid MDE+PTSD. This interpretation is also consistent with models of salience-network dysfunction in PTSD (61) and with visceromotor and interoceptive dysfunction in depression (34), both of which implicate the sgACC and AI in integrating bodily state, affective salience, and stress regulation. Postmortem anatomical evidence identifying AVP-immunoreactive fibers in the ACC and insular cortices further supports the plausibility of vasopressinergic influences on this circuitry (41). In addition, prior resting-state fMRI work has shown that comorbid MDE+PTSD is associated with altered functional connectivity specifically across fear and reward circuitry, including the amygdala and nucleus accumbens (62). These systems are closely integrated with medial prefrontal and frontoinsular regions, including the sgACC (34) and AI (51), suggesting that the abnormalities observed here may reflect a broader systems-level reorganization characteristic of the comorbid phenotype. Within this framework, the observed copeptin-linked alpha alterations may reflect altered integration of bodily stress signals with affective self-referential processing, potentially expressed through changes in salience detection, interoceptive threat monitoring, and network switching.

In addition, the secondary DCM results provide a model-based framework for interpreting possible alterations in effective signaling between these key nodes. Several important findings emerged when using a biophysical model to characterize differences in effective connectivity across groups in the sgACC and AI. Bayesian model selection favored an asymmetric sgACC–AI architecture over both the fully interconnected model and the alternative model specifying the opposite directional hierarchy. In canonical microcircuit formulations of DCM for electrophysiological responses grounded in predictive coding theory (63, 64), feedforward connections are interpreted as ascending signals conveyed by superficial pyramidal cells to spiny stellate cells in a higher region, whereas feedback connections reflect descending influences carried by deep pyramidal cells. Within this framework, the winning model is consistent with a functionally asymmetric hierarchy in which the sgACC provides ascending visceromotor or affective prediction error signals to the AI, and the AI returns descending contextual or predictive influences to the sgACC. Notably, the fully interconnected model was not favored, suggesting that the observed effects were better explained by this directional organization of message passing than by undifferentiated reciprocal coupling or the opposite hierarchical arrangement.

Our second-level analysis identified five parameters governing the MDE+PTSD-by-copeptin interaction, suggesting that copeptin still indexes meaningful differences in cortical dynamics intrinsic to the sgACC and AI. Copeptin was found to interact with membrane capacitance and intrinsic signaling parameters differentially for the MDE+PTSD group. Only in this subgroup, higher copeptin levels were associated with increased membrane capacitance of superficial pyramidal cells, consistent with slower excitatory dynamics, as well as decreased membrane capacitance of inhibitory interneurons, consistent with faster inhibitory responsiveness. Higher copeptin levels were also associated with reduced strength of spiny stellate to superficial pyramidal cell connections within both the sgACC and AI, indicating diminished intra-regional excitatory feedforward drive. Within the sgACC specifically, higher copeptin levels were associated with reduced self-inhibition of inhibitory interneurons, consistent with reduced self-damping of the inhibitory population and greater sensitivity to synaptic input in this region.

Taken together, these model-based findings are consistent with the possibility that higher copeptin within the MDE+PTSD group is associated with reduced excitatory throughput and faster inhibitory timing within the modeled circuit. Such findings are consistent with the vast body of preclinical literature demonstrating that, across brain regions, AVP finetunes neuronal modulation of excitation/inhibition balance by activating inhibitory interneurons (65) and inhibiting pyramidal cells (66–68), and that these cell types are rich in AVP receptors (69). More broadly, the DCM findings provide a plausible circuit-level reason for the observed negative copeptin–alpha association in the MDE+PTSD group. Specifically, it offers a plausible circuit-level account through which higher copeptin may relate to lower alpha power in these regions, given the dependence of alpha oscillations on interneuron-mediated synchrony (28). This is further consistent with growing evidence that neuropeptide systems can shape cortical oscillatory dynamics. For example, intranasal oxytocin (which has complementary and overlapping receptor affinity with AVP) shifts frontal alpha asymmetry during social engagement (70), and intranasal AVP modulates theta-band oscillatory signals during prosocial punishment-avoidance learning (71).

These patterns also cohere with prior literature about AVP neuroanatomy and receptor function. Although vasopressinergic signaling is highly receptor subtype- and context-dependent (20), neuronal structures support vasopressinergic communication from its site of production (the hypothalamic parvocellular nucleus) to cortical regions in order to regulate physiology and behavior (72). AVP-containing fibers have been identified in cingulate and insular cortex in both animals and humans (41). The ACC and insular cortex are key regions within the vasopressinergic network to generate thirst in maintaining body fluid homeostasis (73, 74), which is AVP’s main physiological function. Beyond osmoregulation, exogenously (intranasally) administered AVP modulates many cortical regions, including the (sg)ACC and (anterior) insula, each of which correspond with social-affective-behavioral changes such as social-emotional processing (42, 75), social cooperation (76–79), and social cognition (80, 81). This prior human pharmaco-imaging work supports the plausibility that endogenous vasopressinergic tone indexed by copeptin exerts analogous effects on larger neural networks as well as on local microcircuit dynamics within the sgACC and AI.

Taken together with our prior finding of lower basal AVP secretion indexed by copeptin in the broader parent cohort (14), the current data raise the possibility that this comorbid phenotype is marked not only by altered basal vasopressinergic tone at the larger-cohort level but also by altered neural sensitivity to variation in vasopressin-related tone within the present MEG subset. Because copeptin did not differ significantly across groups in the current sample, the present findings should be understood primarily as evidence of altered copeptin–neural coupling in MDE+PTSD. In the modeled MDE+PTSD subgroup, higher copeptin levels were associated with parameter estimates consistent with a circuit configuration with dampened excitatory throughput and relatively strengthened inhibitory control within the sgACC and AI. At a phenomenological level, this may reflect altered filtering, gating, or contextual regulation of affectively and interoceptively salient information in this diagnostic subgroup. Clinically, such alterations may be relevant to symptom domains commonly seen in depressive-traumatic comorbidity, including hyperarousal, trauma-related distress, disturbed interoceptive processing, and possibly dissociative or suicidal phenotypes. Exploratory models including the MADRS and IES-R as covariates did not materially alter the copeptin-by-diagnosis findings, suggesting that the observed neural signatures were not explained solely by subjective depressive or trauma-related symptom severity in this sample. Although the present study was not powered to test symptom dimensions comprehensively, our findings may invite future investigations of how peripheral stress biology and neural signatures are related to the distinctive phenomenology of depressive-traumatic comorbidity.

A major question about these results is how peripheral (i.e., blood) levels of copeptin as a biological marker of magnocellular AVP neuronal activity can impact neuronal function in the central nervous system (CNS), since peptides such as AVP and copeptin do not cross the blood-brain barrier in sufficient concentrations to affect cortical activity in the sgACC and the AI. Several possibilities are potential explanations: 1) activation of magnocellular AVP neurons may be correlated with co-activation/suppression of stress-specific networks within the CNS; 2) activation of neurohypophyseal magnocellular neurons may cause dendritic release of AVP within the CNS of sufficient magnitude to activate AVP V1a receptors in the cortical areas such as the sgACC and AI (82); 3) axon collaterals from projections of magnocellular AVP neurons to the posterior pituitary project to forebrain areas within the CNS (83); and 4) activation of magnocellular AVP neurons that project to the posterior pituitary may cause co-activation of parvocellular AVP neurons in the hypothalamus that project within the CNS, as has been shown for cholecystokinin activation of pituitary oxytocin secretion (84). These and other possibilities remain to be explored with further investigation.

Several limitations should be considered when interpreting these findings. First, the cross-sectional design precludes causal inference regarding the directionality of associations between copeptin and resting-state neural dynamics. Second, although copeptin is currently the most practical and reliable surrogate of peripheral AVP secretion, it does not constitute a direct measure of central AVP signaling, and cerebrospinal fluid sampling was not feasible in this study. Third, although blood sampling and MEG were generally obtained within a clinically proximal window (mean interval 0.60 days), assessments were separated by as many as seven days in a small percentage of participants. Adjustment for this interval did not materially alter the primary finding; however, copeptin’s *in vivo* kinetics indicate that the present associations should not be interpreted as capturing a single uninterrupted secretion event across both measures. Instead, they are more likely to reflect coupling between peripheral vasopressinergic trait-level tone and resting-state neural dynamics at nearby time points. Future studies should further reduce this interval, as same-day acquisition would provide a stronger test of neuroendocrine-neural coupling. Fourth, subgroup sizes were modest and unevenly distributed, particularly for the MDE+PTSD and HV groups relative to the MDE group, which may have limited power and stability of subgroup-specific estimates.

Biological sex may represent an additional source of heterogeneity in vasopressinergic signaling. Basal copeptin concentrations are generally higher in healthy men than women (85), and experimental AVP administration has produced sex-dependent behavioral and neural effects, including differential modulation of insular and limbic regions involved in stress and salience processing (76, 77). In the present source-level analyses, no significant main effect of sex, sex-by-copeptin, or sex-by-diagnosis-by-copeptin interaction was identified. However, the modest and uneven subgroup sizes substantially limited power to characterize sex-specific copeptin–neural relationships. Accordingly, these null findings should not be interpreted as evidence that the observed associations are equivalent across males and females. Larger and more sex-balanced samples are needed to determine whether the relationships observed here differ by biological sex.

The MDE group was also diagnostically heterogeneous, including individuals with either unipolar and bipolar depressive episodes, and participants varied with regard to their current psychotropic medication exposure. Because lithium and diuretics may influence fluid balance and peripheral AVP-related measures, the primary analysis was repeated after excluding current users of each medication. The interaction remained significant after excluding the four lithium-treated participants. After excluding the five participants taking diuretics, the interaction remained spatially evident in overlapping regions but no longer survived cluster-level correction. Because this exclusion removed four participants from the MDE group but only one from the MDE+PTSD group, the loss of corrected significance may reflect reduced power and altered group composition; however, a contribution of diuretic exposure cannot be excluded. These findings therefore warrant replication in larger samples with more standardized medication exposure and sufficient power to evaluate medication-specific effects.

Finally, resting-state analyses cannot directly establish the symptom-specific or behavioral significance of the observed neural signatures. Similarly, because DCM estimates latent effective connectivity parameters from a specified generative model, these findings should be interpreted as secondary, model-based inferences that are consistent with altered circuit dynamics rather than as direct evidence of cellular physiology or absolute reductions in connectivity.

Overall, our findings raise the possibility that altered copeptin–neural coupling may characterize comorbid MDE+PTSD, but this interpretation remains hypothesis-generating and should be tested in larger and more demographically balanced independent cohorts. Future research should also assess whether the observed relationships differ in PTSD-only, MDD-only, bipolar depression-only, and other trauma-exposed groups. In addition, dimensional approaches may be especially informative for clarifying the clinical relevance of copeptin-linked neural signatures, particularly with respect to hyperarousal, dissociation, interoceptive disturbance, and suicidality. Longitudinal studies are needed to determine whether these MEG signatures track clinical state or change with treatment, or whether they predict treatment response over time. Incorporating task-based MEG or experimental stress-challenge paradigms may further clarify how vasopressinergic signaling influences salience- and interoception-relevant circuit function under behaviorally relevant conditions. Finally, multimodal neuroendocrine profiling—including cortisol, adrenocorticotropic hormone, corticotropin-releasing hormone, oxytocin, and Neurophysin-I (as a surrogate for oxytocin secretion)—could help determine whether copeptin identifies a specific vasopressin-related mechanism or serves as part of a broader stress-responsive biotype with translational relevance for biomarker-informed stratification.

## Data Availability

The raw data supporting the conclusions of this article will be made available by the authors, without undue reservation.
