# Supplementary material for: Plasma copeptin is coupled to resting-state frontoinsular-subgenual alpha dynamics in major depression with comorbid post-traumatic stress disorder
Source: Front Endocrinol (Lausanne). 2026 Sep 3;17:1895698. doi: 10.3389/fendo.2026.1895698 (PMC13581851; doi:10.3389/fendo.2026.1895698)
Supplement: Supplementary file 1 [file Supplementaryfile1.docx]

*Hu et al — Plasma Copeptin is Coupled to Resting-State Frontoinsular-Subgenual Alpha Dynamics in Major Depression with Comorbid Post-Traumatic Stress Disorder*

**SUPPLEMENT**

**Participant Recruitment and Selection from the Parent Clinical Protocol**

Participants in the present study were drawn from the ongoing “Neurobiology of Suicide” protocol (NCT02543983), a National Institute of Mental Health study designed to characterize clinical, biological, and neuroimaging features associated with suicidal thoughts and behaviors across a range of psychiatric presentations. Adults aged 18–70 years were recruited from the community and underwent comprehensive screening before enrollment. Psychiatric diagnoses were established using the Structured Clinical Interview for DSM-5 Disorders (SCID-5-CT) [1].

All participants provided written informed consent, and the protocol was approved by the National Institutes of Health Institutional Review Board. Safety procedures for conducting research involving participants with suicide risk have been described previously [2].

The parent protocol was designed to enroll participants across a spectrum of suicide risk, including healthy volunteers (HVs) without psychiatric history and clinical participants with mood and anxiety symptoms of varying severity. For the present study, the subset of selected participants had: (1) available baseline plasma copeptin measurements, (2) baseline resting-state magnetoencephalography (MEG) data, and (3) diagnostic information permitting classification into one of three analytic groups: HVs, participants experiencing a current major depressive episode (MDE) without post-traumatic stress disorder (PTSD) (the MDE group), and participants experiencing a current MDE with comorbid PTSD (MDE+PTSD).

HVs were required to have no current or past psychiatric disorder. Clinical participants were required to meet criteria for a current MDE, either in the context of major depressive disorder or bipolar disorder, and were categorized according to the presence or absence of comorbid PTSD. Because the parent study was not originally designed around PTSD comorbidity as a primary grouping variable, the analytic groups in the present report were derived retrospectively from the structured diagnostic assessment.

General exclusion criteria for non-HV participants in the parent protocol included current psychotic features, cognitive impairment or other conditions compromising the ability to provide informed consent, current substance dependence or acute intoxication, unstable medical illness, clinically significant laboratory abnormalities, and pregnancy or breastfeeding. Individuals whose acute suicide or homicide risk could not be safely managed within the research setting were also excluded. Additional details regarding parent-protocol procedures and safety monitoring have been reported elsewhere [2].

The present report focuses exclusively on baseline clinical, biomarker, and resting-state MEG data and does not include interventional procedures.

**Supplemental References**

[1] W.J. First MB, Karg RS, Spitzer RL, Structured Clinical Interview for DSM-5 Disorders, Clinical Trials Version (SCID-5-CT). American Psychiatric Association, Arlington, VA, 2015.

[2] E.D. Ballard, L. Waldman, J.S. Yarrington, N. Gerlus, L.E. Newman, L. Lee, M. Sparks, V. Liberty, M. Pao, L. Park, and C.A. Zarate, Jr., Neurobiological research with suicidal participants: A framework for investigators. Gen Hosp Psychiatry 62 (2020) 43-48.

**Supplementary Table S1. Distribution of the absolute interval between blood collection and magnetoencephalography (MEG) acquisition**

| Absolute interval, days | Participants, n | Sample, % |
| --- | --- | --- |
| 0 (same day) | 67 | 63.8 |
| 1 | 31 | 29.5 |
| 2 | 2 | 1.9 |
| 3 | 0 | 0.0 |
| 4 | 1 | 1.0 |
| 5 | 1 | 1.0 |
| 6 | 2 | 1.9 |
| 7 | 1 | 1.0 |
| Total | 105 | 100.0 |

The interval is reported as the absolute number of days between blood sampling and resting-state MEG acquisition, regardless of which assessment occurred first. Sixty-seven participants (63.8%) completed both assessments on the same day, and 98 (93.3%) completed them within one day. The median absolute interval was 0 days (interquartile range, 0-1), and the mean (SD) was 0.60 (1.25) days.

**Supplementary Figure S1: Alpha diagnosis-by-copeptin interaction.**

Brain regions demonstrating a group-by-copeptin interaction in the alpha (8–14 Hz) frequency are superimposed on a template brain when removing participants taking lithium (top) and diuretics (bottom). The interaction survived cluster-level correction when removing participants taking lithium but not those taking diuretics.
